# Transient Receptor Potential Mucolipin-1 Channels in Glioblastoma: Role in Patient’s Survival

**DOI:** 10.3390/cancers11040525

**Published:** 2019-04-12

**Authors:** Maria Beatrice Morelli, Consuelo Amantini, Daniele Tomassoni, Massimo Nabissi, Antonella Arcella, Giorgio Santoni

**Affiliations:** 1 School of Pharmacy, University of Camerino, 62032 Camerino, Italy; mariabeatrice.morelli@unicam.it (M.B.M.); massimo.nabissi@unicam.it (M.N.); 2 School of Biosciences and Veterinary Medicine, University of Camerino, 62032 Camerino, Italy; consuelo.amantini@unicam.it (C.A.); daniele.tomassoni@unicam.it (D.T.); 3 IRCCS NEUROMED, 86077 Pozzilli, Italy; arcella@neuromed.it

**Keywords:** glioblastoma, TRP channel, TRPML-1, mucolipins, autophagy, overall survival

## Abstract

A link between mucolipin channels and tumors has been recently suggested. Herein, we aim to investigate the transient receptor potential mucolipin (TRPML)-1 relevance in glioblastoma. The expression of this channel was evaluated via qRT-PCR and immunohistochemistry in biopsies from 66 glioblastoma patients and two human glioblastoma cell lines and compared to normal human brain, astrocytes, and epileptic tissues. The subcellular distribution of TRPML-1 was examined via confocal microscopy in the glioma cell lines. Then, to assess the role of TRPML-1, cell viability assays have been conducted in T98 and U251 cell lines treated with the specific TRPML-1 agonist, MK6-83. We found that MK6-83 reduced cell viability and induced caspase-3-dependent apoptosis. Indeed, the TRPML-1 silencing or the blockage of TRPML-1 dependent [Ca^2+^]_i_ release abrogated these effects. In addition, exposure of glioma cells to the reactive oxygen species (ROS) inducer, carbonyl cyanide m-chlorophenylhydrazone (CCCP), stimulated a TRPML-1-dependent autophagic cell death, as demonstrated by the ability of the autophagic inhibitor bafilomycin A, the TRPML-1 inhibitor sphingomyelin, and the TRPML-1 silencing to completely inhibit the CCCP-mediated effects. To test a possible correlation with patient’s survival, Kaplan–Meier, univariate, and multivariate analysis have been performed. Data showed that the loss/reduction of TRPML-1 mRNA expression strongly correlates with short survival in glioblastoma (GBM) patients, suggesting that the reduction of TRPML-1 expression represents a negative prognostic factor in GBM patients.

## 1. Introduction

Glioblastoma (GBM) is the most aggressive and prevalent type of glioma, with a median overall survival (OS) of 12–15 months [1,2]. Although new therapeutic options have been developed on the basis of new knowledge about the molecular nature of GBM, surgery in association with radiation therapy and chemotherapy remains the standard of care. Several reports demonstrated the important role played by ion channels belonging to the transient receptor potential (TRP) superfamily in GBM [3,4].

Among the TRP family, mucolipins (TRPML channels) represent a distinct subfamily of endosome/lysosome Ca^2+^ channel proteins [5]. In mammals, there are three TRPML proteins (TRPML-1, -2, and -3) encoded by *MCOLN1-3* [6]. With regards to human, TRPML-2 is expressed in astrocytes and neural stem/progenitor cells. We have recently demonstrated the overexpression of TRPML-2 in high-grade GBM cell lines of astrocytic origin and GBM tissues [7]. Knockdown of TRPML-2 inhibits cell viability and proliferation and induces caspase-3-dependent apoptosis in GBM cell lines [7].

At present, no data on the expression and function of TRPML-1 in GBM tissues and cell lines have been provided. *MCOLN-1* located on human chromosome 19 [8] was identified as the gene mutated in human Mucolipidosis type IV (MLIV), a progressive neurodegenerative disease of children [9,10,11]. TRPML-1 is ubiquitously expressed in mammalian cells and it is localized primarily in the late endosome/lysosome [12,13,14]. It consists of six transmembrane helices, two pore helices, and a luminal 25 kDa domain [15]. In addition, it has a large intraluminal loop between its first and second transmembrane domains that contains a putative serine-lipase site, a proline-rich domain, and a proteolytic cleavage site [11]. This loop may interact with chaperone-mediated autophagy-related proteins such as the heat shock cognate protein of 70 kDa (Hsc70), and the 40-kDa heat shock protein (Hsp40) [16]. TRPML-1 has been also found to target the *Apoptosis-linked gene-2* (*ALG-2*), also known as *programmed cell death 6* (*PDCD6*), which codifies for ALG-2, an EF-hand-containing protein promoting caspase-3-independent-cell death, associated to GBM progression and poor prognosis [17,18].

TRPML-1 is a proton-impermeable, cation-selective channel with permeability to both Ca^2+^ and Fe^2+^. It is ligand-gated and is activated by phosphatidylinositol-3,5-biphosphate (PtdIns(3,5)P2), voltage, extracellular or luminal low pH as well as by MK6-83 and ML-SA1 synthetic compounds [19,20,21], whereas it is inhibited by phosphatidylinositol-4,5-biphosphate (PtdIns(4,5)P2), sphingomyelins, verapamil, lysosomal adenosine, and mammalian target of rapamycin kynase (mTOR) kinase [21,22,23,24,25]. 

The functions of TRPML proteins include roles in vesicular trafficking and biogenesis, maintenance of neuronal development, lysosome integrity, and regulation of intracellular and organellar ionic homeostasis. TRPML-1 plays a role in the control of cell viability and in chaperone-mediated autophagy [16]. It is involved in death of mammalian cells induced by lysosomotropic agents [26]. TRPML-1 is considered a reactive oxygen species (ROS) sensor localized on the lysosomal membrane that orchestrates an autophagy-dependent negative-feedback program to mitigate oxidative cell stress [27]. In addition, TRPML-1 forms homo- and hetero-multimers with TRPML-2 and/or TRPML-3 as well as with the two-pore channels (TPCs) (e.g., TPC1 and TPC2) [28,29] that seem to play a crucial role in regulating cell viability and starvation-induced autophagy [30,31].

In the present work, we investigated the expression and the function of TRPML-1 channels in GBM cell lines. Moreover, the correlation between the TRPML-1 expression and GBM patients’ overall survival has been also evaluated.

## 2. Results

### 2.1. TRPML-1 Expression in T98 and U251 GBM Cell Lines

**Figure 1:**
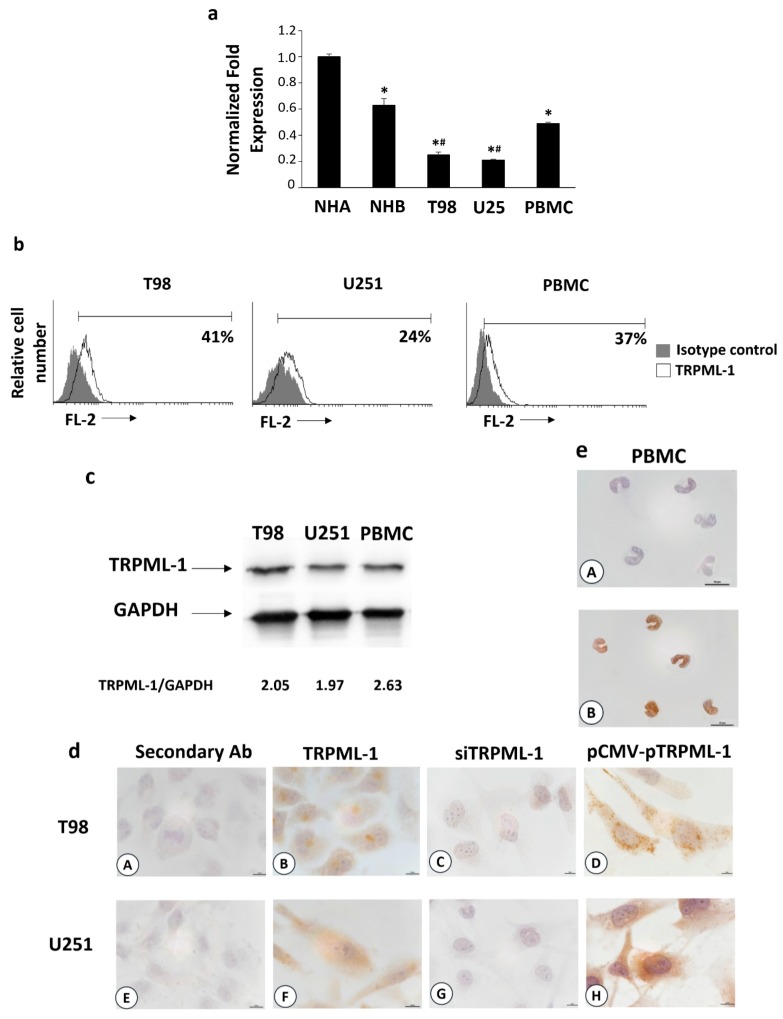
Transient receptor potential mucolipin (TRPML)-1 expression in glioblastoma (GBM) cell lines. (**a**) The relative TRPML-1 mRNA expression in normal human astrocytes (NHA), normal human brain (NHB), T98, and U251 glioma cell lines, and in peripheral blood mononuclear cells (PBMCs) used as positive control were evaluated by qRT-PCR. TRPML-1 mRNA levels were normalized for glyceraldehyde-3-phosphate dehydrogenase (GAPDH) expression. Data are expressed as mean ± SD. * *p* < 0.05 vs. NHA; # *p* < 0.05 vs. NHB, PBMCs. (**b**) Flow cytometric analysis was performed in GBM cells, fixed, permeabilized, and stained with anti-human TRPML-1 Ab followed by phycoerythrin (PE)-conjugated secondary Ab. Isotype control Ab was used as negative control. Numbers represent the percentage of TRPML-1 positive cells. (**c**) Total lysates were separated on 8% SDS-PAGE and probed with anti-TRPML-1 and anti-GAPDH Abs. Blots are representative of one of three separate experiments. Numbers represent the densitometric analysis as compared with GAPDH. (**d**) Immunocytochemical stains for TRPML-1 in untransfected (B,F), siTRPML-1 (C,G), and pCMV-pTRPML-1 (D,H) glioma cell lines. Scale bar: 10 μm. (**e**) Immunocytochemical stain for TRPML-1 in PBMC (A,B). Scale bar: 10 μm. Cells were formaldehyde-fixed, permeabilized, probed with anti-human TRPML-1 Ab, and biotinylated anti-mouse IgG1, ABC reagent, and substrate solution containing DAB. Nuclei were stained with hematoxylin. Representative images are shown. The incubation with the secondary antibody alone was used as negative control (**d**A, **d**E, **e**A). Scale bar: 10 μm.

TRPML-1 mRNA expression was evaluated in human T98 and U251 GBM cell lines by qRT-PCR. Its expression was observed in both cell lines, although at lower levels compared to normal human astrocytes (NHA, *n* = 2), normal human brain (NHB, *n* =2), and peripheral blood mononuclear cells (PBMCs) used as positive controls (Figure 1a) [9]. By cytofluorimetric and fluorescence-activated cell sorting (FACS) analysis data showed that about 41% and 24% of T98 and U251 cells express TRPML-1 protein (Figure 1b). Immunoblots from T98 and U251 glioma cell lysates incubated with anti-TRPML-1 antibody (Ab) F-10 clone showed a band corresponding to human TRPML-1 (Figure 1c). Similar results were obtained using the specific anti-TRPML-1 Ab MLN128 clone. Furthermore, by immunocytochemistry, TRPML-1 reactivity was evidenced in both T98 and U251 cell lines (Figure 1d). PBMCs were used as positive control (Figure 1e). TRPML-1 knockdown in both glioma cell lines was used as negative control (Figure 1d). Silencing experiments were performed by RNA interference. At first, by qRT-PCR and western blot analysis, we evaluated the efficacy of gene silencing. TRPML-1 mRNA and protein levels were decreased by about 70% in cells silenced for TRPML-1 (siTRPML-1) with respect to the transfection control cells (siGLO) at 48 and 72 h post-transfection, respectively (Figure S1a,b). Immunocytochemistry confirmed no reactivity in both silenced cell lines (Figure 1d). However, to further support the expression results, glioma cell lines were transiently transfected with a Mammalian Expression Vectors containing CicloMegaloVirus promoter upstream (pCMV) encoding the full-length coding sequence of *TRPML-1*. mRNA and protein expression in pCMV-pTRPML-1 with respect to pCMV (transfection control) glioma cells was evaluated by qRT-PCR and western blot analysis. After 48 h of transfection, TRPML-1 mRNA levels increased by about 60% (Figure S1c), and after 72 h, protein levels were confirmed higher in pCMV-pTRPML-1 with respect to pCMV T98 and U251 transfected glioma cells (Figure S1d). Noteworthily, in this transfection model, immunocytochemistry evidenced a strong nuclear and perinuclear reactivity (Figure 1d).

### 2.2. Subcellular Expression of TRPML-1 in Glioblastoma Cell Lines

**Figure 2:**
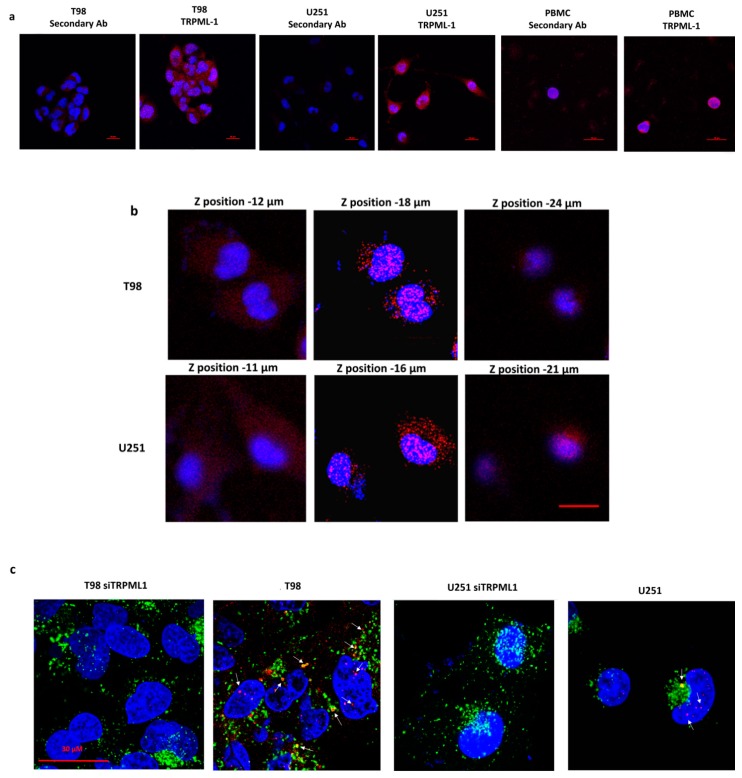
Subcellular distribution of TRPML-1 in glioblastoma cell lines. Cells were fixed, permeabilized, and stained with anti-human TRPML-1 Ab followed by Alexa Fluor-594 secondary Ab. 4′,6-diamidino-2-phenylindole (DAPI) was used to counterstain nuclei. (**a**) Confocal microscopy analysis of TRPML-1 expression in glioma cells and PBMC, used as positive control. Calibration bar: 20 μm. (**b**) Z-Stack of glioma cells, stained as described above was performed using confocal microscopy. Pictures were taken on several planes, ranging from upper to lower levels. Calibration bar: 20 μm. (**c**) Colocalization with endolysosomal compartment was analyzed by staining untransfected and siTRPML-1 transfected cells with anti-LAMP-1 Ab, followed by incubation with Alexa Fluor-488 secondary Ab. Calibration bar: 30 μm.

Immunocytochemistry results prompted us to examine the subcellular distribution of TRPML-1 in glioma cell lines by confocal laser scanning microscopy. As shown in Figure 2a, TRPML-1 localized mainly in the cytoplasm with a clustered pattern in PBMCs, while in T98 and U251 cell lines TRPML-1 was expressed as dot spots in the cytoplasmic and nuclear compartments (Figure 2a). Thanks to Z-axis analysis, we further demonstrated the TRPML-1 punctuate distribution in the nucleus of these cells and in perinuclear position (Figure 2b). Thus, to better appreciate the TRPML-1 protein localization, we performed a double staining using an Ab against human lysosomal-associated membrane protein (LAMP)-1, an endolysosomal marker. As shown in panel c, TRPML-1 can be localized to both nucleus and endolysosomes (Figure 2c). TRPML-1-silenced cell lines were used as negative control.

**Figure 3:**
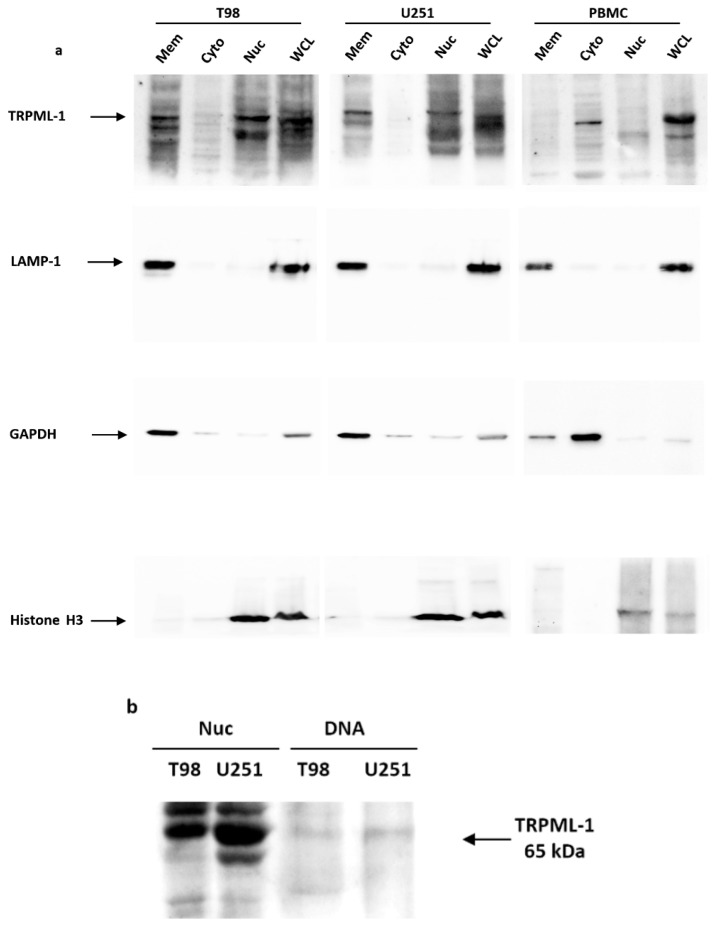
TRPML-1 nuclear localization in glioblastoma cell lines. (**a**) Proteins derived from membrane fraction (Mem), cytosolic fraction (Cyto), nuclear/cytoskeletal fraction (Nuc), and whole cell lysate (WCL) were immunoblotted with anti-TRPML-1 Ab. Whole cell lysate was used as control. The purity of subcellular fractions was assessed by blotting against specific markers. Cytosolic and membrane marker: GAPDH; membrane-bound organelles markers: LAMP-1; nuclear marker: Histone H3. Blots are representative of one of three separate experiments. (**b**) To analyze the ability of TRPML-1 to bind DNA, nuclear fraction (Nuc) proteins and DNA were isolated from T98 and U251. The samples were electrophoresed in SDS-PAGE gel and incubated with anti-TRPML-1 Ab to determine the relative protein expression. Data are representative of three separate experiments.

Data were confirmed by western blot and protein-DNA binding analyses. The TRPML-1 localization in GBM cell lines was evaluated in membrane, cytosolic, nuclear T98, U251, and PBMC fractions (Figure 3a). Whole cell lysates (WCL) were used as control, while LAMP-1, glyceraldehyde-3-phosphate dehydrogenase (GAPDH), and Histone H3 were used to check the subcellular fraction separation. In both GBM cell lines, TRPML-1 appeared to be localized in the nucleus and in membrane/organelle fractions positive for LAMP-1, whereas it appeared to be not expressed in the cytoplasmic fraction. Nuclear localization was further confirmed by Histone H3 positivity in nuclear extracts. Regarding PBMC used as control, TRPML-1 is mainly expressed in the cytoplasm. 

TRPML-1 nuclear localization was further investigated through protein-DNA binding assay and western blot analysis (Figure 3b), in order to examine TRPML-1 DNA-binding ability. The analysis was conducted on nuclear fraction proteins and DNA isolated from T98 and U251 cell lines; total nuclear fraction was used as control. The samples were then electrophoresed in SDS-PAGE gel and, finally, blotted with mouse anti-human TRPML-1 Ab. A band of about 65 kDa, likely corresponding to the TRPML-1 protein, was evidenced in T98 and U251 cells nuclear lysates, confirming TRPML-1 DNA-binding ability.

### 2.3. The Specific TRPML-1 Agonist, MK6-83, Triggers Intracellular Ca^2+^ Rise and Inhibits the Viability in T98 and U251 Cells

**Figure 4:**
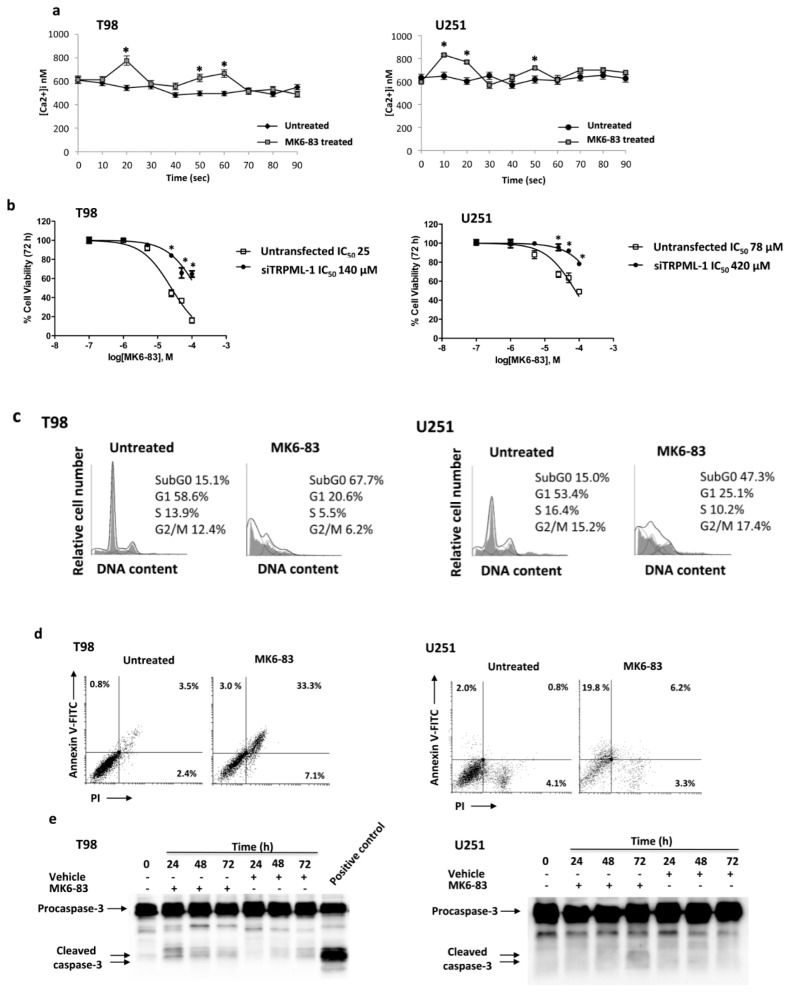
MK6-83 induces TRPML-1 activation and triggers T98 and U251 apoptotic cell death. (**a**) Time course of the [Ca^2+^]_i_ rise was evaluated by FACS analysis in T98 and U251 GBM cells untreated or treated with 10 μM and 25 μM of MK6-83, respectively. Data shown are the mean ± SD of three independent experiments. Statistical analysis was determined by comparing MK6-83-treated with untreated cells, * *p* < 0.05. (**b**) Cell viability was evaluated by 3-(4,5-dimethylthiazol-2-yl)-2,5-diphenyltetrazolium bromide (MTT) assay in untransfected or TRPML-1-silenced (siTRPML-1) T98 and U251 GBM cells treated with different doses of MK6-83 for 72 h. Data shown are expressed as mean ± SE of three separate experiments. (**c**) Representative cell cycle distribution in GBM cells treated for 72 h with MK6-83 10 μM in T98 and 25 μM in U251 cells. Data are one out of three separate experiments. (**d**) Biparametric flow cytometric analysis was performed in T98 and U251 cells, untreated or treated with MK6-83 for 48 h, by Annexin V- Fluorescein isothiocyanate (FITC) and Propidium iodide (PI) staining. Cells in the upper left quadrant indicate Annexin V-positive, early apoptotic cells. The cells in the upper right quadrant indicate Annexin V-positive/PI-positive, late apoptotic cells. (**e**) Lysates from T98 and U251 cells, untreated or treated with MK6-83 for different times, and from positive control for caspase-3 activation were separated on SDS-PAGE and probed with anti-caspase-3 Ab. Blots are representative of three separate experiments.

Activation of TRPML channels induces Ca^2+^ release [30], thus we performed a dose response assay to evaluate [Ca^2+^]_i_ levels in glioma cells stimulated with a TRPML-1 specific agonist. At present, none of the currently available TRPML agonists are selective and specific for TRPML-1. T98 and U251 have been found to express TRPML-2 [7], so the agonist ML-SA1 that activates all three human TRPML isoforms cannot be utilized. MK6-83 has been found to activate both TRPML-1 and TRPML-3 [21,32]. Thus, we firstly evaluated the expression of TRPML-3 in NHA, GBM tissues, GBM cell lines, and myeloma multiple (MM) cell lines used as positive control. No TRPML-3 transcript was found in NHA cells and GBM cells and tissues, whereas it was evidenced in MM cell lines (Figure S2). These results prompted us to use MK6-83 to selectively stimulate TRPML-1 in glioma cells. Treatment with MK6-83 at 10 μM in T98 and 25 μM in U251 cells induced [Ca^2+^]_i_ rise in both Ca^2+^ free medium-treated glioma cell lines with respect to untreated cells, suggesting that the TRPML-1 channel is functional and promotes Ca^2+^ release from intracellular stores (Figure 4a). Silenced glioma cells were used as negative control model for calcium release (Figure S3).

To evaluate the effect of TRPML-1 in glioma cell viability, MTT assays on T98 and U251 cells have been performed. A dose-dependent reduction in cell viability was evidenced in both MK6-83-treated compared to vehicle-treated cells after 72 h culture (Figure 4b). Noteworthily, T98 cells were more sensitive than U251, showing an IC_50_ value of 25 μM compared to 78 μM of U251 cells. To confirm the TRPML-1 involvement in MK6-83 effects, TRPML-1 silencing was performed in both glioma cell lines and cell viability was analyzed after 72 h of MK6-83 treatment. TRPML-1 silencing markedly reduced the MK6-83-induced growth inhibition, with an increase of IC_50_ from 25 to 140 μM and from 78 to 420 μM in T98 and U251, respectively (Figure 4b). 

### 2.4. TRPML-1 Activation Triggers Caspase-Dependent Apoptosis in T98 and U251 Cells

Cell cycle analysis was performed to evaluate the effect of TRPML-1 activation treating glioma cells with MK6-83 at sub-optimal doses: 10 μM for T98 and 25 μM for U251. The TRPML-1 agonist strongly reduced the percentage of cells in G1 phase and increased that in subG0 phase at 72 h post treatment, indicating the presence of an elevated percentage of hypodiploid cells with fragmented DNA in both cell lines, compared with untreated cells (Figure 4c). Therefore, the capability of the MK6-83 to induce cell death was evaluated by Annexin V-Fluorescein isothiocyanate (FITC)/ Propidium iodide (PI) staining and cytofluorimetric analysis. Results showed that MK6-83 induces apoptosis in both glioma cell lines, although with different kinetics. Indeed, at 48 h post treatment, 30% of T98 cells were Annexin V-positive/PI-positive (late apoptosis), while 18% of U251 cells were in Annexin V-positive/PI-negative (early apoptosis) (Figure 4d). These data were confirmed by western blot analysis showing that TRPML-1 activation in T98 and U251 cells induces caspase-3 cleavage at 24 and 72 h after MK6-83 treatment, respectively (Figure 4e). Moreover, dose-response experiments further support these results showing an increase of caspase-3 cleaved form with increased doses in T98 after 24 h and in U251 after 72 h of treatment (Figure S4). No LC3-I to LC3-II conversion was evidenced in MK6-83-treated T98 and U251 cells, suggesting that TRPML-1 activation by MK6-83 did not induce autophagy (Figure S5). Moreover, by dichlorodihydrofluorescein diacetate (DCFDA) staining and cytofluorimetric analysis, no ROS production was found in MK6-83-treated T98 and U251 cells, at different time after treatment. 

**Figure 5:**
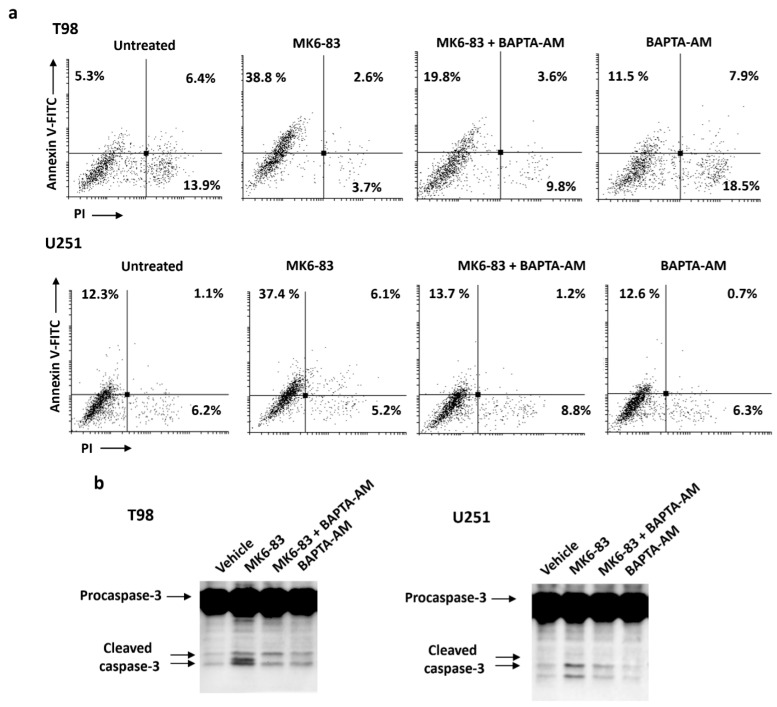
The effect of intracellular Ca^2+^ chelator 1,2-Bis(2-aminophenoxy)ethane-N,N,N′,N′-tetraacetic acid tetrakis (acetoxymethyl ester) (BAPTA-AM) on intracellular events associated with MK6-83-induced apoptosis in glioma cells. (**a**) BAPTA-AM (10 μM) was applied 2 h before the addition of MK6-83 for 24 h in T98 and for 48 h in U251. Biparametric flow cytometric analysis was performed by Annexin V-FITC and PI staining. (**b**) Lysates from T98 and U251 cells pretreated with BAPTA-AM and then treated with MK6-83 for 24 h in T98 and for 72 h in U251 were separated on SDS-PAGE and probed with anti-caspase-3 Ab. Blots are representative of three separate experiments.

To examine the role of intracellular calcium in MK6-83-induced apoptosis, the effects of intracellular calcium chelator 1,2-Bis(2-aminophenoxy)ethane-N,N,N′,N′-tetraacetic acid tetrakis (acetoxymethyl ester) (BAPTA-AM) on cell death and cleavage of caspase-3 were studied. Glioma cells were pretreated with BAPTA-AM (10 μM) for 2 h before exposure to MK6-83 for up to 72 h. After 24 h of cotreatment, BAPTA-AM significantly reduced MK6-83-induced apoptotic cell death, as evaluated by Annexin V/PI staining. In both cell lines, there is about 50% of Annexin V-positive cells reduction in cotreated with respect to MK6-83-alone-treated cells (Figure 5a). Moreover, via immunoblot, we demonstrated that the cotreatment with BAPTA-AM in T98 after 24 h and in U251 after 72 h attenuates the MK6-83-induced caspase-3 cleavage compared to MK6-83-treated cells (Figure 5b). Since BAPTA-AM alone did not interfere with apoptosis, our data indicate that intracellular Ca^2+^ is involved in the MK6-83-induced apoptotic processes in glioma cells. 

### 2.5. The ROS Inducer, Carbonyl Cyanide m-Chlorophenylhydrazone (CCCP), Triggers TRPML-1-Dependent Autophagic Cell Death in GBM Cell Lines

**Figure 6:**
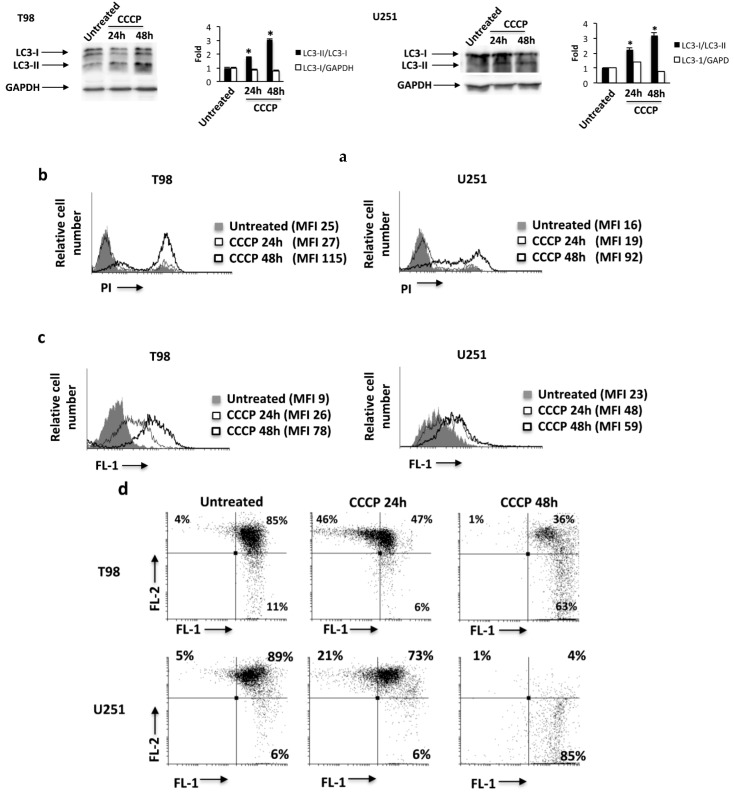
The carbonyl cyanide m-chlorophenylhydrazone (CCCP) exposure triggers reactive oxygen species (ROS) production, mitochondrial depolarization, and autophagy in T98 and U251 cells. (**a**) Lysates from T98 and U251 cells, untreated or treated for 24 h and 48 h with CCCP, were separated on 14% SDS-PAGE and probed with anti-LC3 and anti-GAPDH Abs. GAPDH protein levels were evaluated as loading control. Blots are representative of one of three separate experiments. Bars represent the densitometric analysis. * *p* < 0.05 vs. untreated cells. (**b**) PI incorporation was analyzed by flow cytometry in T98 and U251 cells treated as described above. Histograms are representative of one of three separate experiments. MFI = mean fluorescence intensity. (**c**) To analyze ROS production in GBM cells, treated as described above, were stained with dichlorodihydrofluorescein diacetate (DCFDA) before flow cytometric analysis. Histograms are representative of one of three separate experiments. (**d**) T98 and U251 cells were treated with CCCP as described above and the mitochondrial transmembrane potential (ΔΨm) changes were evaluated by tetraethylbenzimidazolylcarbocyanine iodide (JC-1) staining and biparametric FL-1(green)/FL-2(red) flow cytometric analysis. Data are representative of one out of three separate experiments.

Autophagy plays an important role in cellular response to oxidative stress [33,34] and the role of TRPML-1 as cellular stress sensor has been previously described [27,35]. Since mitochondria are the primary source of endogenous reactive oxygen species (ROS), we exposed glioma cells for 24 h and 48 h to the mitochondrial respiration inhibitor, carbonyl cyanide m-chlorophenylhydrazone (CCCP, 10 μM), commonly used to induce ROS production, mitochondrial damage, and mitophagy [27]. Increased conversion of the LC3-I in the LC3-II lipidated form was found at 24 and mainly at 48 h after CCCP exposure in T98 and U251 cells, indicating that CCCP induces autophagy of these cell lines (Figure 6a). 

Moreover, cell death, ROS production, as well as the mitochondrial potential were measured by PI, DCFDA, and tetraethylbenzimidazolylcarbocyanine iodide (JC-1) staining and cytofluorimetric analysis in GBM cells treated with CCCP. Results showed that treatment with CCCP at 48 h increases the PI fluorescence (Figure 6b), enhances ROS production (Figure 6c), and markedly reduces mitochondrial transmembrane potential (ΔΨm) (Figure 6d). Neither Annexin V-positive cells nor caspase 3 activation was evidenced in CCCP-treated glioma cells. 

**Figure 7:**
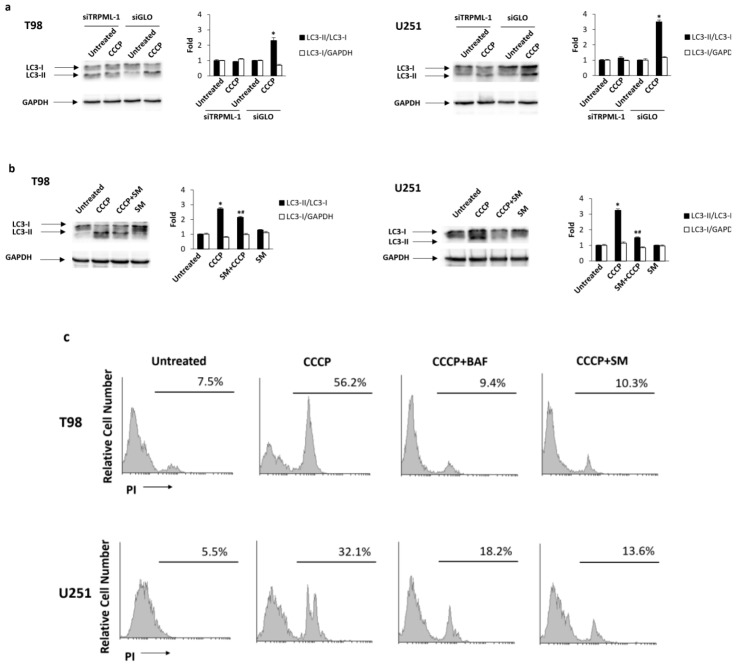
CCCP induces TRPML-1-dependent autophagic cell death in T98 and U251 cells. (**a**) Lysates from siTRPML-1 and siGLO T98 and U251 cells treated for 48 h with CCCP, were separated on 14% SDS-PAGE and probed with anti-LC3 and anti-GAPDH Abs. GAPDH protein levels were evaluated as loading control. Blots are representative of one of three separate experiments. Bars represent the densitometric analysis. * *p* < 0.05 vs. untreated cells. (**b**) Lysates from T98 and U251 cells, pretreated for 1 h with sphingomyelin (SM) and treated for 48 h with CCCP, were separated by SDS-PAGE and probed with anti-LC3 and anti-GAPDH Abs. GAPDH protein levels were evaluated as loading control. Blots are representative of one of three separate experiments. Bars represent the densitometric analysis. * *p* < 0.05 vs. untreated and SM; # *p* < 0.05 vs. CCCP. (**c**) The cytotoxic effects in T98 and U251 cells, pretreated with SM or bafilomycin A (BAF) before the addition of CCCP, were determined by PI staining and cytofluorimetric analysis assay. A representative of three experiments has been shown.

To further investigate the role of TRPML-1 in CCCP-induced autophagy, TRPML-1-silenced glioma cells were treated with CCCP for 48 h. As shown in Figure 7a, while in siGLO control cells, CCCP increased the conversion of LC3-I in LC3-II, while in the siTRPML-1 cells it was not able to (Figure 7a). Moreover, we also evaluated the effects of the TRPML-1 inhibitor, sphingomyelin (SM) [21]. The pretreatment with 20 μM SM for 1 h inhibited CCCP-induced autophagy in both T98 and U251 cell lines, suggesting that the CCCP-induced autophagy is TRPML-1 mediated (Figure 7b). SM alone did not influence LC3 conversion (Figure 7b). 

Since autophagy can mediate pro-survival or pro-death functions, we stained glioma cells treated for 48 h with CCCP alone or in combination with 50 nM bafilomycin A (BAF), with PI and performed cytofluorimetric analysis. As shown in Figure 7c, BAF completely reverted the CCCP-induced cell death, demonstrating that CCCP promoted an autophagic cell death. In addition, to understand the role of TRPML-1, T98 and U251 cells pretreated with SM and then exposed to CCCP were analyzed by PI staining and cytofluorimetric analysis. SM markedly reduced the percentage of PI-positive cells indicating that CCCP-induced autophagic cell death is TRPML-1 dependent (Figure 7c). 

Overall, these results suggested that in glioma cells, TRPML-1, functioning as an oxidative stress sensor, induces the activation of autophagy in order to promote cell death.

### 2.6. TRPML-1 as Prognostic Factor in GBM Patients

**Figure 8:**
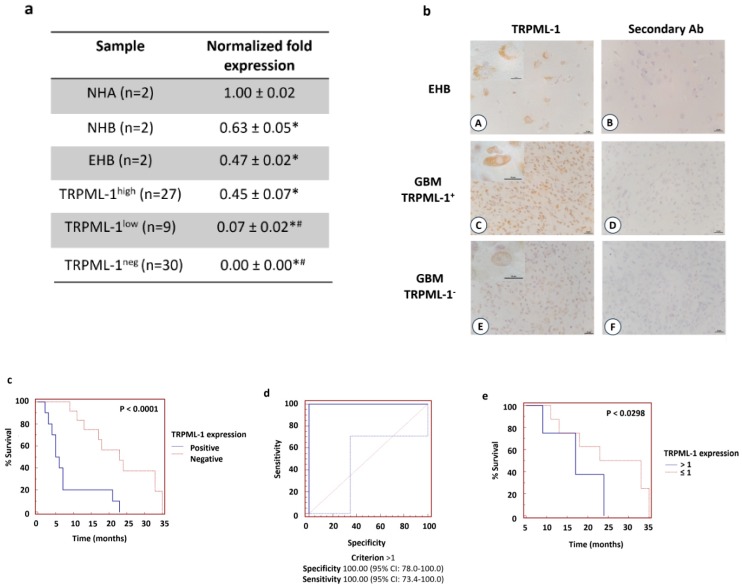
Loss of TRPML-1 expression correlates with poor prognosis in GBM patients. (**a**) The relative TRPML-1 mRNA expression in NHA, NHB, epileptic human brain (EHB), and GBM samples was evaluated by qRT-PCR. TRPML-1 mRNA levels were normalized for GAPDH expression. Data are expressed as mean ± SD. * *p* < 0.05 vs. NHA, # *p* < 0.05 vs. NHB and EHB. (**b**) Immunohistochemical analysis of TRPML-1 protein in human EHB tissues (A,B) and GBM (C–F). Sections from paraffin-embedded tissues were stained with anti-human TRPML-1 Ab, biotinylated anti-mouse IgG1, ABC reagent, and substrate solution containing DAB. Nuclei were stained with hematoxylin. Sections incubated with the omission of primary Ab were used as negative control (B,D,F). Calibration bar: 25 μm. Inserts’ scale bar: 10 μm. (**c**) Kaplan–Meier plot for GBM patients in TRPML-1^+^ and TRPML-1^-^ groups. (**d**) ROC analysis of TRPML-1 mRNA expression in the TRPML-1+ GBM patients (*n* = 36). (**e**) Kaplan–Meier plot for GBM patients stratified according to ROC analysis.

The expression of TRPML-1 was evaluated at mRNA levels in human GBM tissues (*n* = 66) (Table S1), NHA (*n* = 2), or NHB (*n* = 2) total mRNA and in non-tumor epileptic human brain (EHB) samples (*n* = 2). About 54.5% (*n* = 36) of GBM tissues express, although at lower level respect to NHA, NHB, or EHB samples (Figure 8a), TRPML-1 mRNA, whereas 45.5% (*n* = 30) of the samples were TRPML-1 negative. 

TRPML-1 expression was also analyzed at protein levels by immunohistochemistry. Similar to qRT-PCR analysis, TRPML-1 immunoreactivity was evidenced in 36 GBM patients and in EHB tissues, used as positive control. In EHB samples, only neurons developed immunoreaction at the level of the cytoplasm with perinuclear localization (Figure 8b). In GBM tissues, cells develop immunoreaction with a different degree of intensity (Figure 8b). No reactivity was present in tissue sections incubated with the omission of the primary Ab. 

Then, we calculated the mean and the median OS of GBM patients. We found that the mean OS was 14.4 months and the median OS was 11.0 months. By Kaplan–Meier method, we evaluated the correlation between patients’ OS and TRPML-1 mRNA expression in TRPML-1^+^ (*n* = 36) and TRPML-1^-^ (*n* = 30) GBM patients (*n* = 66). The median OS of TRPML-1^-^ patients was significantly shorter than that of TRPML-1^+^ (5.5 months vs. 23 months; *p* < 0.0001, HR = 3.8734, 95% CI 4.2433–16.8156) (Figure 8c). Concordantly, via univariate analysis, a statistically significant difference in OS was evidenced between TRPML-1^+^ and TRPML-1^-^ GBM patients (*p* < 0.0001, 95% CI 0.01938–0.4045). Moreover, by subgrouping TRPML-1^+^ GBM patients according to ROC analysis (Figure 8d) in TRPML-1 > 1, TRPML-1 ≤ 1 the OS were of 28 and 17 months, respectively (*p* < 0.0298, HR = 2.2018, 95% CI 1.1221–9.4147) (Figure 8e). Additionally, we evaluated, via multivariate Cox regression analysis, the correlation between the expression of TRPML-1, O-6-methylguanine-DNA methyltransferase (MGMT), and adjuvant therapy with OS in GBM patients. No significant differences were found for MGMT (*p* = 0.2333) and adjuvant therapy (*p* = 0.3210), whereas TRPML-1 maintains statistical significance for survival (*p* < 0.0235).

In conclusion, low or absent TRPML-1 expression strongly correlates with short survival in GBM patients, suggesting that the reduction of TRPML-1 mRNA expression represents a negative independent prognostic factor in GBM patients.

## 3. Discussion

Recently, a link between endolysosomal mucolipin channels and cancer has been suggested [36]. At this regard, we have previously reported that overexpression of TRPML-2 mucolipin channel promotes glioma progression [7]. Herein, we evaluated the expression of TRPML-1 in GBM. Our results demonstrated that TRPML-1 mRNA and protein is variably expressed in GBM with samples showing lower expression compared to NHA, NHB, and EHB, and others showing a complete loss of TRPML-1. Similarly, low TRPML-1 levels were observed in T98 and U251 glioma cell lines, compared to NHA. Previously, it has been reported that in normal cells, TRPML-1 protein is expressed in the late endosome/lysosome. Instead, by confocal microscope and western blot analysis of subcellular fractions, we found that in both glioma cell lines, it localizes in endolysosomes and as dot spots in the nuclear cell compartment. Similar results regarding TRPM2, another member of the TRP family, have been obtained in prostate cancerous cell lines and in breast adenocarcinomas. In these tumor cell lines, TRPM2 is located in the nuclei, whereas in non-cancerous prostate BPH-1 and HMEC and MCF-10A human mammary epithelial cells, it is localized in the plasma membrane and in the cytoplasm [37,38]. Which are the mechanisms in tumor cells responsible for nuclear localization? The TRPML-1 protein contains a nuclear localization signal (NLS), an amino-acid sequence that allows the import into the cell nucleus [10,39]; other mechanisms, such as phosphorylation within or proximal to the NLS sequence of a cargo protein as well as different conformational state of TRPML-1 protein or homo and heteromerization, may be responsible for nuclear localization. What are the consequences of this altered localization? In *C. elegans* when the C-terminal fragment of the TRPV-1 channel is translocated into the nucleus, it alters the endogenous transcription of a wide variety of genes [40]. In our study, we presented, for the first time, the TRPML-1 ability to bind DNA somehow, although whether it affects the transcription of genes controlling tumor survival and progression is not yet understood and further studies must be performed. 

**Figure 9:**
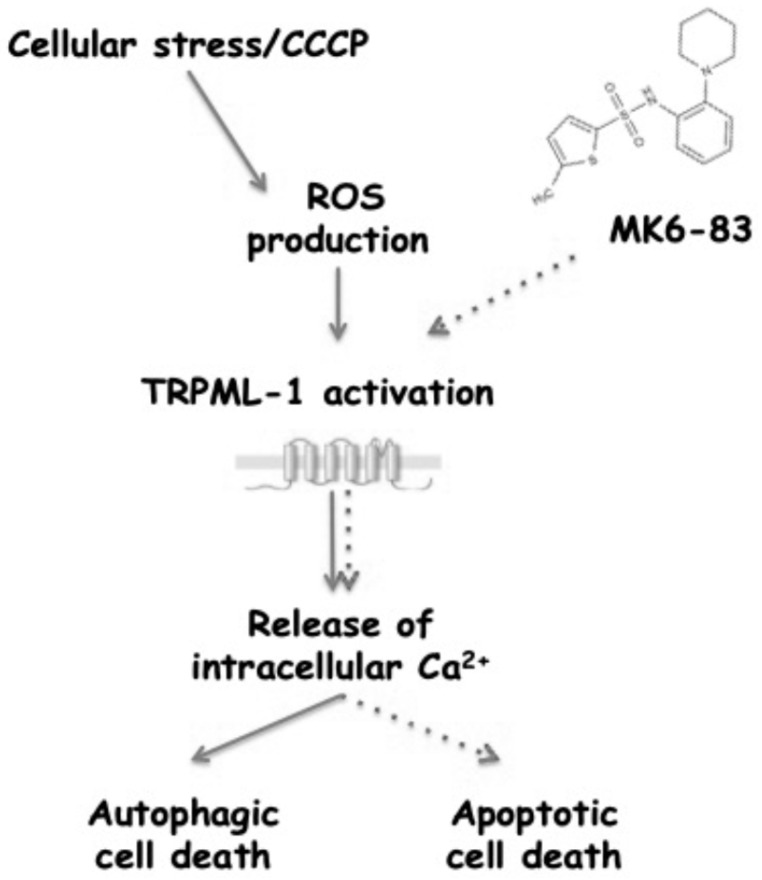
Schematic representation of TRPML-1-mediated cytotoxic effects.

Overall, the role of TRPML-1 in GBM has been evaluated by using MK6-83, a potent activator of the TRPML-1 channel [20]. Treatment of GBM cell lines with the agonist reduced viability and induced caspase-dependent apoptotic cell death, and these effects were abrogated by the specific TRPML-1 knocking down. Neither ROS production nor autophagic activation was triggered by MK6-83 treatment, in accordance with Zhang’s report [27]. Autophagy represents the front line of defense against oxidative stress in both normal and neoplastic cells [34]. Mounting evidences revealed that mitochondria, the major site of endogenous ROS production, could modulate the autophagy process [34]. In cancers, autophagy can be stimulated in response to ROS injury and mitochondrial ROS may function as molecular switch for regulating autophagic fate [34]. A TRPML-1 role in starvation- and oxidative stress-induced autophagy has been also reported [37,41]. Increased ROS levels activate TRPML-1, leading to lysosomal Ca^2+^ release and enhancement of autophagy by PPP3/calcineurin-dependent TFEB nuclear translocation [35,42]. The mitochondrial decoupler CCCP is able to induce TRPML-1-dependent calcium currents [27], thus, to better understand the role of TRPML-1 as oxidative stress sensor, we exposed GBM cells to this compound. CCCP-inducing ROS production stimulates autophagic cell death in GBM cells. Noteworthily, TRPML-1 silencing as well as the pretreatment with SM, a specific inhibitor of TRPML-1 activity, reverted the CCCP effects. Our data in GBM cells are in agreement with Zhang and coworkers’ findings showing a role of TRPML-1 as ROS sensor in oxidative-stress-induced autophagy [27]. Therefore, TRPML-1-mediated autophagy seems to require two different signals (Figure 9): Ca^2+^ rise, which stimulates autophagosome biogenesis, and ROS production, which promotes lysosome biogenesis [43]. In our models, we take advantage of the stressor CCCP to indirectly activate TRPML-1 and induce both Ca^2+^ increase and ROS generation, thus triggering autophagic cell death. In contrast, the direct TRPML-1 activation by its agonist MK6-83 induces Ca^2+^ release but no ROS production, thus inducing apoptosis.

Finally, the correlation between patients’ survival and TRPML-1 mRNA expression was, for the first time, evaluated in TRPML-1-positive vs. TRPML1-negative GBM patients. The expression of TRPML-1 mRNA reached high significance for survival, with TRPML-1-expressing GBM patients showing a more favorable OS. These results were further strengthened subgrouping the TRPML-1^+^ GBM patients according to ROC analysis. We found that the OS of GBM patients was gradually lowered at the reduction of the TRPML-1 mRNA value. Thus, low or absent TRPML-1 expression strongly correlates with short survival in GBM patients, suggesting that the loss/reduction of TRPML-1 mRNA expression represents a negative prognostic factor in GBM patients. On the other hand, the TRPML-1 expression exerts a protective survival effect in GBM patients. 

We are only at the beginning; further studies are required to completely understand the physio-pathological role of TRPML-1 and the other members of mucolipin ion channel family in tumor development and progression. 

## 4. Materials and Methods

### 4.1. Cells and Tissues

Formalin-fixed paraffin-embedded brain tissues from human tumor biopsies and epileptic brain (EHB) (*n* = 2) surgically removed from patients who gave informed consent to the study (*n* = 66) were kindly provided by Prof. Felice Giangaspero (I.N.M., Neuromed, Pozzilli, Isernia, Italy). Glioblastoma tissues (grade IV) were histologically graded according to the World Health Organization classification criteria [2]. Total RNAs from normal human brain (NHB, two different batches) and normal human astrocytes (NHA, two different batches) were purchased from DBA (Milan, Italy). 

The glioblastoma T98 and U251 cell lines (grade IV), obtained from European Collection of Cell Cultures (ECACC, Salisbury, UK), were maintained in Dulbecco’s modified Eagle’s medium (DMEM, Lonza Bioresearch, Basel, Switzerland) supplemented with 10% heat inactivated fetal bovine serum (FBS), 2 mmol/L L-glutamine, 100 IU/mL penicillin, 100 μg streptomicin at 37 °C, 5% CO_2_, and 95% humidity. 

Peripheral blood mononuclear cells (PBMC) were isolated from the human peripheral blood of healthy donors. Blood was diluted with Dulbecco’s phosphate buffered saline (PBS) without calcium and magnesium (Lonza Bioresearch) (ratio 1:1). The diluted samples were subjected to density gradient separation on Ficoll Paque Plus (ratio 1:1) (GE Healthcare Life Sciences, Buckinghamshire, UK), centrifuged, and collected.

### 4.2. Chemical and Reagents

3-(4,5-dimethylthiazol-diphenyltetrazolium bromide (MTT), propidium iodide (PI, 2 µg/mL), ribonuclease A (100 μg/mL), dichlorodihydrofluorescein diacetate (DCFDA, 20 μM), the TRPML-1 agonist MK6-83 (solubilized in DMSO), the TRPML-1 inhibitor sphingomyelin (SM, 20 µM; solubilized in methanol:chloroform 1:1), carbonyl cyanide m-chlorophenylhydrazone (CCCP, 10 µM; solubilized in DMSO), and BAPTA-AM (10 µM) were purchased from Sigma Aldrich (Milan, Italy). Bafilomycin A (BAF, 50 nM; solubilized in DMSO) was from Invitrogen (Toulouse, France). 5,5′,6,6′-tetrachloro-1,1′,3,3′-tetraehylbenzimidazolylcarbocyanineiodide (JC-1, 10 µg/mL) was purchased from Life Technologies (Italia, Italy). Annexin V-FITC from Enzo Life Sciences (Farmingdale, NY, USA). The following rabbit polyclonal antibodies (Abs) were used: Anti-caspase-3 (1:1000, Cell Signaling Technology, Danvers, MA, USA), anti-microtubule-associated protein-1 light chain 3 (LC3, 2 μg/mL, Novus Biologicals, Littleton, CO, USA), and anti-Histone H3 (1:1000, Cell Signaling Technology). The following mouse monoclonal Abs were used: Anti-TRPML-1 (clone F-10, Santa Cruz Biotechnology (Dallas, TX, USA): 1:300 for western blot, 1:25 in immunohistochemistry and immunofluorescence, 1:50 for FACS analysis), anti-TRPML-1 (clone MLN128, Sigma Aldrich: 3 μg/mL for western blot, 1:25 in immunohistochemistry and immunofluorescence; 1:50 for FACS analysis), anti-LAMP-1 (1:300, Santa Cruz Biotechnology), and anti-glyceraldehyde-3-phosphate dehydrogenase (anti-GAPDH, 14C10, 1:1000, Cell Signaling Technology). The following secondary antibodies were used: Horseradish peroxidase (HRP)-conjugated anti-mouse IgG and HRP-conjugated anti-rabbit IgG (1:2000, Cell Signaling Technology); biotinylated anti-rabbit IgG and biotinylated anti-mouse IgG (1:200, Bethyl, Montgomery, TX, USA); FITC-conjugated goat anti-mouse Ab (BD Biosciences, Milan, Italy), Alexa Fluor-594-conjugated goat anti-mouse Ab (1:100; Invitrogen, San Diego, CA, USA), Alexa Fluor-488-conjugated goat anti-mouse Ab (1:100; Invitrogen).

### 4.3. Western Blot Analysis

To obtain whole cell lysate, cells were lysed in a lysis-buffer containing protease inhibitor cocktail (EuroClone, Milan, Italy). Cytoplasmatic, membrane/organelle, and nuclear/cytoskeletal fractions were isolated using the Cell Fractionation Kit (Cell Signaling Technology) according to the manufacturer’s instruction.

Proteins were separated on 8–14% SDS polyacrylamide gel in a Mini-PROTEAN Tetra Cell system (BioRad, Hercules, CA, USA). Protein transfer from the gel to a nitrocellulose membrane was carried out using Mini Trans-Blot Turbo RTA system (BioRad). 

Non-specific binding sites were blocked with 5% low-fat dry milk and 2% bovine serum albumin (BSA) in phosphate-buffered saline 0.1% Tween 20 for 1 h at room temperature. Membranes were incubated overnight at 4 °C in primary Abs (anti-caspase 3, anti-LC3, anti-TRPML-1, anti-LAMP-1, anti-Histone H3, and anti-GAPDH), followed by the incubation for 1 h at room temperature with HRP-conjugated anti-rabbit or anti-mouse secondary Abs. 

The detection was performed using the LiteAblot PLUS or Turbo kits (EuroClone), and densitometric analysis was carried out by a Chemidoc using the Quantity One software (version 4.6.7, BioRad). For quantification, GAPDH was used as loading control. One representative out of three independent experiments is shown in each immunoblot figure.

### 4.4. Protein-DNA Binding Assay

Protein-DNA Binding Assay was performed using EPItech ChIP One day kit (Qiagen, Milan, Italy) following manufacturing protocol. For each assay, chromatin from about 3 × 10^6^ cells was fragmented to an average size from about 500 to 1500 bp by eight rounds of sonication (Power: 0.5 W, Time: 2 s on, 15 s off; total time 16 s) in 2 mL tubes using the Sonicator 3000 (MISONIX, Part # 3000) (QSonica, LLC, Newtown, CT, USA). The nuclear/DNA fraction was used to analyze the presence of TRPML-1 by western blot analysis.

### 4.5. TRPML-1 Transfection Models

For silencing experiments, TRPML-1 (siTRPML-1) and siCONTROL non-targeting siRNA (siGLO, used as negative control) FlexiTube siRNA were purchased from Qiagen (Milan, Italy). For gene silencing experiments, T98 and U251 cell lines were plated at the density of 1.2 × 10^5^/mL and siTRPML-1 or siGLO (150 ng for T98, 75 ng for U251) was added to the wells, following the HiPerfect transfection reagent transfection protocol (Qiagen). No differences were observed comparing siGLO transfected with untransfected cells.

For overexpression experiments, glioma cells were plated at a density of 1.2 × 10^5^/mL. After overnight incubation, transfections were achieved with 7.5 μL/mL of the reagent TransIT-X2 (Mirus MIR-6003, OriGene, Rockville, MD, USA) and 2.5 μg/mL of pCMV-pTRPML-1 or pCMV empty (pCMV) vectors according to the manufacturer’s instructions (Origene, Castenaso, Italy). No differences were observed comparing pCMV transfected with untransfected cells.

### 4.6. MTT Assay

Three × 10^4^/mL untreated, siGLO, or siTRPML-1 glioma cells were plated in 96-well plates and treated with different doses of MK6-83 up to 72 h. Then, 0.8 mg/mL of MTT was added to the samples and incubated for additional 3 h. After the removal of medium from the wells, the formazan crystals were dissolved with 100 μL per well of DMSO and the colored solutions were read by microtiter plate spectrophometer (BioTek Instruments, Winooski, VT, USA). Four replicates were used for each treatment. IC_50_ values, showed as mean ± standard error (S.E.), correspond to the drug concentration that induces 50% of cell growth inhibition compared to control cells. IC_50_ values were calculated using GraphPad Prism^®^ 5.0a (GraphPad Software, San Diego, CA, USA).

### 4.7. Calcium Mobilization Assay

For calcium influx analysis, cells were resuspended in medium supplemented with 7 μmol/L FLUO 3-AM (Invitrogen) and 1 μg/mL Pluronic F-127 (Invitrogen) and incubated in the dark for 30 min at 37 °C and 5% CO_2_. FLUO 3-AM fluorescence was measured by FACS [44]. [Ca^2+^]_i_ was determined before and after the addition of MK6-83 in medium without adding Ca^2+^. The following equation was used to determine [Ca^2+^] free: [Ca^2+^] free = Kd[F−Fmin]/[Fmax−F], where kd of Fluo 3 is 400 nM, F is the sample mean fluorescence, Fmax is obtained by exposing the cells to ionomycin, and Fmin is evaluated by exposing ionomycin-treated cells to manganese chloride. Unstimulated cells were analyzed to establish baseline fluorescence levels.

### 4.8. Cell Cycle Analysis

For cell cycle analysis, MK6-83-treated T98 and U251 cells were fixed in ice-cold 70% ethanol, treated for 30 min at 37 °C with 100 μg/mL ribonuclease A solution, stained for 30 min at room temperature with PI 20 μg/mL, and analyzed by flow cytometry using linear amplification.

### 4.9. Mitochondrial Transmembrane Potential (ΔΨm)

Mitochondrial transmembrane potential was evaluated by JC-I staining in CCCP-treated T98 and U251 cells at 24 h and 48 h after treatment. Cells were incubated for 10 min at room temperature with JC-1. JC-I was excited by an argon laser (488 nm) and green (530 nm)/red (>570 nm) emission fluorescence was collected simultaneously. Samples were analyzed by a FACScan cytofluorimeter using the CellQuest software (version 5.1, Beckton Dickinson, San Jose, CA, USA); the fluorescence intensity was expressed in arbitrary units on logarithmic scale.

### 4.10. ROS Production

The fluorescent probe DCFDA was used to assess oxidative stress levels in CCCP-treated T98 and U251 cells at 24 h and 48 h after treatment. Cells were incubated with 20 μM DCFDA 20 min prior to the harvest time point. The cells were then washed, and the intensity of the fluorescence was assayed using flow cytometry and CellQuest software.

### 4.11. Cell Death Analysis

Cell death was evaluated using FITC-conjugated Annexin V and PI staining followed by single and biparametric FACS analysis. Briefly, treated cells were incubated with 5 μL Annexin V-FITC and 20 μg/mL PI for 10 min at room temperature. In some experiments, cells were pretreated with SM, BAF, or BAPTA-AM, and then CCCP or MK6-83 was added for an additional 24/48 h. At the end of the experiment, cells were stained with PI. The cells were then analyzed by flow cytometry using CellQuest software.

### 4.12. Gene Expression Analysis

Total RNA from fixed paraffin-embedded tissue slices (5–7 μm thick) was extracted by RNeasy^®^ FFPE Mini Kit (Qiagen, Milan, Italy). RNA from U251 and T98 cell lines was extracted by RNeasy Mini Kit (Qiagen). All RNA samples were eluted in the appropriate buffer and their concentration and purity were evaluated by A260/280 nm measurement. In total, 800 ng of RNA extracted were subjected to reverse transcription in a total volume of 20 μL using the iScript kit (BioRad) according to the manufacturer’s instructions. Then, 5 μL of the cDNA derived by fixed paraffin-embedded tissue was pre-amplified for 15 cycles using SsoAdvanced PreAmp Supermix kit (BioRad). One microliter of the resulting cDNA products were used as template for quantitative real-time polymerase chain reaction (qRT-PCR).

Quantitative RT-PCR was performed by using the IQ5 Multicolor real-time PCR detection system (BioRad). The reaction mixture contained the Advanced Universal SYBRGreen Supermix (BioRad). Human TRPML-1 and GAPDH RT2 qPCR Primer assay (Qiagen) were used. The PCR parameters were 10 min at 95 °C followed by 40 cycles at 95 °C for 15 s and 60 °C for 40 s. All samples were assayed in triplicate in the same plate. The relative amount of target mRNA was calculated by the 2^−ΔΔCt^ method, using GAPDH as a housekeeping gene.

### 4.13. Immunohistochemistry

For immunohistochemistry, after re-hydration, tissue sections were incubated with Tris-HCl 20 mM, EDTA 0.65 mM, Tween-20 0.05% pH 9, in a microwave for 5 min (two times) for antigen retrieval. Sections were treated with H_2_O_2_ for 20 min, washed, incubated for 1 h at room temperature with 5% bovine serum albumin (BSA) and 0.3% Triton X-100 in PBS, and then overnight at 4 °C with anti-TRPML-1 Ab. Thereafter, slides were incubated for 30 min at room temperature with a biotinylated secondary Ab, rinsed, and exposed for 30 min to the streptavidin–biotin complex (VECTASTAIN ABC Kit, Vector laboratories, Burlingame, CA, USA). Immunoreactivity was detected by the addition of 3,3′-diaminobenzidine (DAB, Peroxidase Substrate Kit, Vector laboratories, Burlingame, CA, USA). Four random fields of each tissue specimen were analyzed under ×40 magnification using the Leica DMR Microscope collected by TV camera (Nikon DS-Ri2) with an NIS Element Imaging Software (Nikon Instruments, Firenze, Italy). 

Untransfected, siGLO, siTRPML-1, pCMV, and pCMV-pTRPML-1 T98 and U251 cells were maintained on 8-well culture slides in fresh medium. After 72 h, cells were fixed with 2% and 4% paraformaldehyde with 0.5% of Triton X-100 in PBS for 10 min at room temperature and were then counterstained with hematoxylin. Immunohistochemistry in these slides was performed as described above, without antigen-retrieval.

### 4.14. Confocal Laser Scanning Microscopy Analysis

Untransfected, siGLO, and siTRPML-1 T98 and U251 cells were maintained on 8-well culture slides in fresh medium, fixed, and permeabilized using 2% and 4% of paraformaldehyde with 0.5% of Triton X-100 in PBS. After washes in PBS, cells were incubated with 5% of bovine serum albumin (BSA) and 0.1% of Tween-20 in PBS for 1 h at room temperature and then stained with anti-TRPML-1 Ab overnight at 4 °C. Then, samples were washed with 0.3% of Triton X-100 in PBS and incubated with Alexa Fluor 594-conjugated secondary Ab for 1 h at 37 °C. In colabeling experiments, cells were also stained with anti-LAMP-1 Ab overnight at 4 °C. Finally, samples were washed with 0.3% of Triton X-100 in PBS and incubated with Alexa Fluor 488-conjugated secondary Ab for 1 h at 37 °C. Nuclei were stained with DAPI. Slides were then analyzed with C2 Plus confocal laser scanning microscope (Nikon Instruments, Firenze, Italy). Optimized emission detection bandwidths were configured by Zeiss Zen control software. Z-stack acquisition intervals were selected to satisfy Nyquist sampling criteria. Images were processed using NIS Element Imaging Software (Nikon Instrumentes, Firenze, Italy).

### 4.15. Immunofluorescence and FACS Analysis

Cells were fixed with 4% paraformaldehyde and then stained with anti-TRPML-1 Ab or normal mouse IgG1 isotype control (Santa Cruz Biotechnology) in permeabilization buffer (PBS, 1% FBS, 0.1% NaN_3_ and 1% saponin). After an incubation of 1 h at 4 °C, cells were then incubated with FITC-conjugated secondary Ab and analyzed using a FACScan cytofluorimeter with CellQuest software.

### 4.16. Statistical Analysis

The statistical significance was determined by Student’s *t*-test and by ANOVA with Bonferroni’s post-test. Overall survival was defined as the interval between the date of surgery to death or last follow-up visit. Median overall survival (OS) was estimated using Kaplan–Meier method with Rothman’s 95% confidence intervals (CI) and compared across the groups using the log-rank test. For univariate analysis of significance, the long-rank test or Cox analysis was used. The Cox proportional hazards model was used for multivariate analysis. *p* < 0.05 was considered as statistically significant. On the basis of the TRPML-1 mRNA levels, the GBM patients were divided in TRPML-1^+^ and TRPML-1. These groups were subjected to survival analysis. Moreover, Kaplan–Meier analysis was performed in TRPML-1^+^ GBM patients (*n* = 36) stratified in TRPML-1 > 1, TRPML-1 ≤ 1 according to ROC analysis. Statistical analysis was performed with MedCalc package (MedCalc^®^ version 16.4.3, Ostend, Belgium).

## 5. Conclusions

Overall, our data demonstrated that the reduction of TRPML-1 expression represents a negative prognostic factor in GBM patients. They could be particularly relevant in diagnosis, prognosis, and therapy of GBMs. Indeed, on the basis of the TRPML-1 mRNA levels, it could be possible to stratify different GBM patients showing short, intermediate, or long OS, suggesting TRPML-1 as a new target for a different therapeutic approach in TRPML-1-expressing GBM patients. 

## Supplementary Materials

The following are available online at https://www.mdpi.com/2072-6694/11/4/525/s1, Figure S1: TRPML-1 silenced and overexpressed models, Figure S2: TRPML-3 expression in GBM cell lines, Figure S3: Intracellular calcium measurements in silenced glioma cells, Figure S4: MK6-83 induces caspase-3 cleavage in a dose-dependent manner, Figure S5: MK6-83 does not induce autophagy in GBM cell lines. Table S1: GBM (grade IV) patient characteristics.
