# Supplementary material for: Transient Receptor Potential Mucolipin-1 Channels in Glioblastoma: Role in Patient’s Survival
Source: Cancers (Basel). 2019 Apr 12;11(4):525. doi: 10.3390/cancers11040525 (PMC6521337; doi:10.3390/cancers11040525)
Supplement: Supplementary file 1 [file cancers-11-00525-s001.pdf]

Supplementary Materials: Transient Receptor Potential Mucolipin-1 Channels in Glioblastoma: Role in Patient's Survival

Maria Beatrice Morelli, Consuelo Amantini, Daniele Tomassoni, Massimo Nabissi, Antonella Arcella and Giorgio Santoni

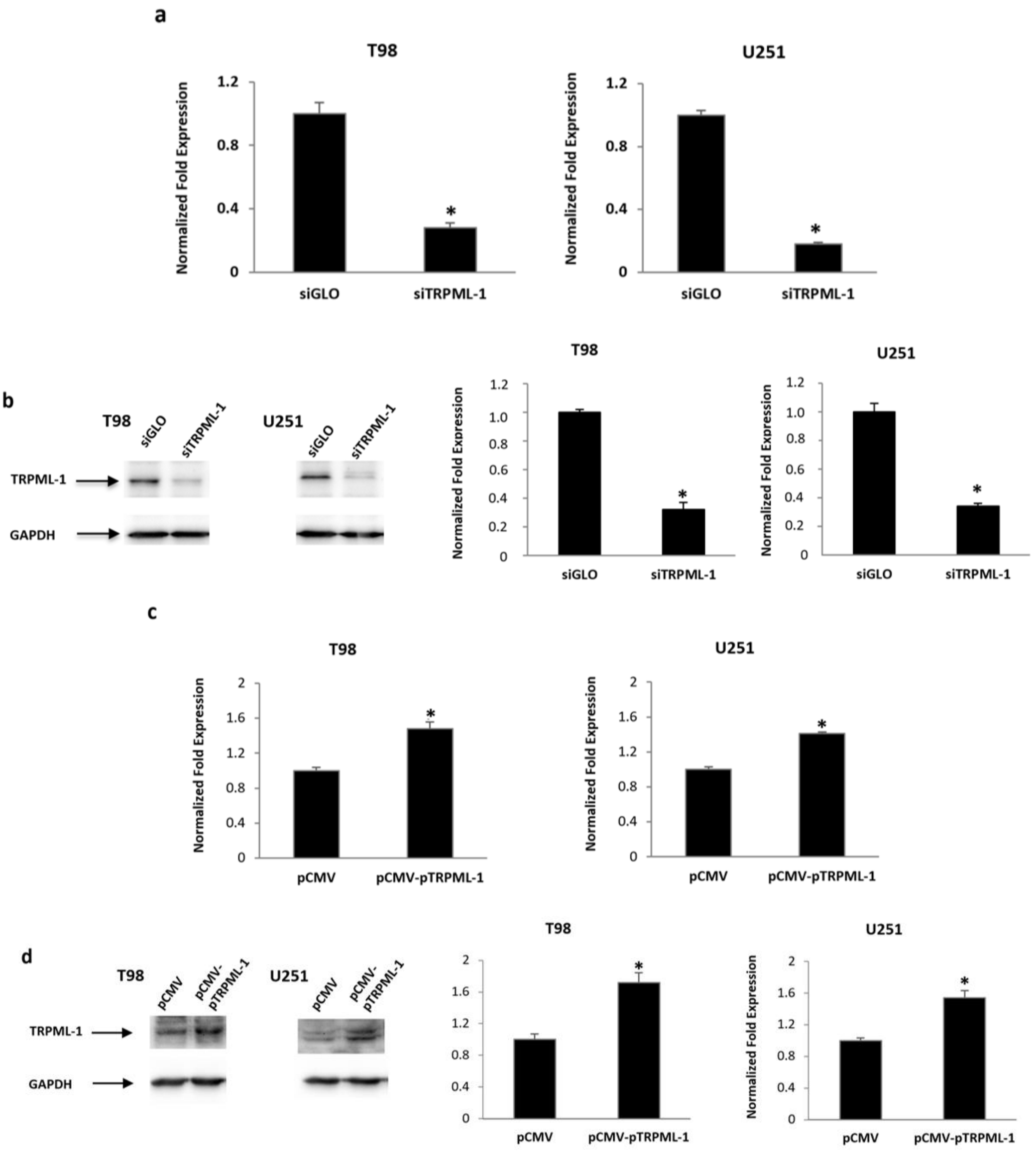

Figure S1. TRPML-1 silenced and overexpressed models. (**a**) TRPML-1 mRNA levels were evaluated by qRT-PCR in siTRPML-1 and siGLO T98 and U251 cells after 48 h of transfection. Relative TRPML-1 expression, normalized to GAPDH mRNA levels, was calculated using siGLO as calibrator. Statistical analysis was performed comparing siTRPML-1 with siGLO transfected cells, * $p < 0.01$. (**b**) Lysates from siGLO and siTRPML-1 T98 and U251 transfected cells were separated on SDS-PAGE and probed with anti-human TRPML-1 Ab. GAPDH protein levels were evaluated as loading control. Relative TRPML-1 expression values after 72 h of transfection were calculated using siGLO as calibrator. Blots are representative of three separate experiments. * $p < 0.01$ vs. siGLO. (**c**) TRPML-1 mRNA levels were evaluated by qRT-PCR in pCMV and in pCMV-pTRPML-1 T98 and U251 cells after 48 h of transfection. Relative

TRPML-1 expression, normalized to GAPDH mRNA levels, was calculated using pCMV as calibrator. Statistical analysis was performed comparing pCMV-pTRPML-1 with pCMV transfected cells, * $p < 0.01$. (**d**) Lysates from pCMV and pCMV-pTRPML-1 T98 and U251 transfected cells were separated on SDS-PAGE and probed with anti-human TRPML-1 Ab. GAPDH protein levels were evaluated as loading control. Relative TRPML-1 expression values after 72 h of transfection were calculated using pCMV as calibrator. Blots are representative of three separate experiments. * $p < 0.01$ vs. pCMV.

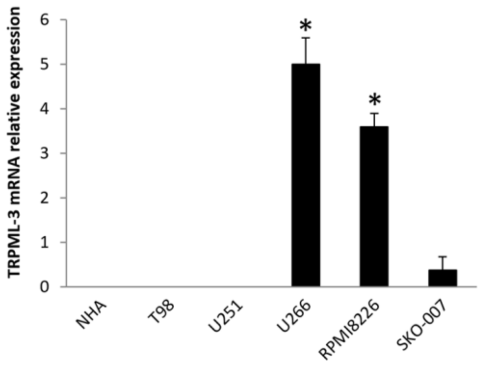

Figure S2. TRPML-3 expression in GBM cell lines. The relative TRPML-3 mRNA expression in NHA, T98 and U251 glioma cell lines, and in U266, RPMI8226 and SKO-007 myeloma multiple cell lines used as positive controls was evaluated by qRT-PCR. TRPML-3 mRNA levels were normalized for GAPDH expression. Data are expressed as mean ± SD. * p <0 .05 vs. NHA, T98, U251 and SKO-007.

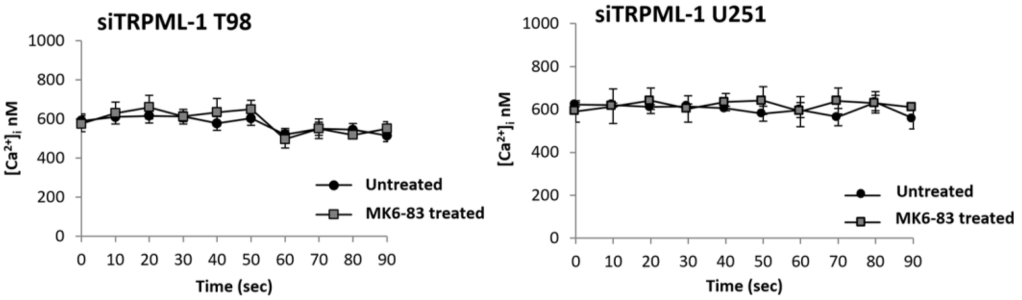

Figure S3. Intracellular calcium measurements in silenced glioma cells. Time course of the $[Ca^{2+}]_i$ rise was evaluated by FACS analysis in siTRPML-1 T98 and U251 GBM cells untreated or treated with 10 μM and 25 μM of MK6-83, respectively. Data shown are the mean ± SD of three independent experiments.

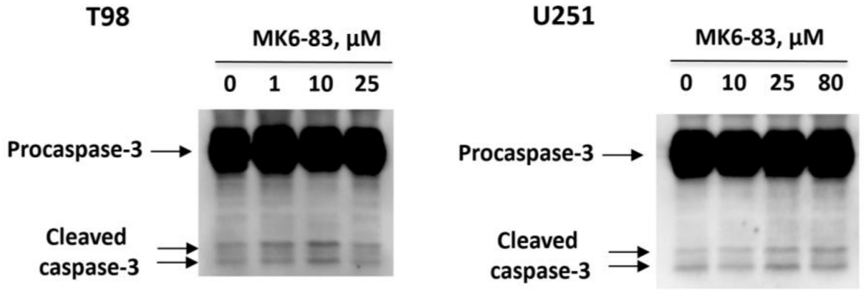

Figure S4. MK6-83 induces caspase-3 cleavage in a dose-dependent manner. T98 and U251 cells were treated with different doses of MK6-83. Proteins were separated on 14% SDS-PAGE and probed with anti-caspase-3 Ab. Blots are representative of three separate experiments.

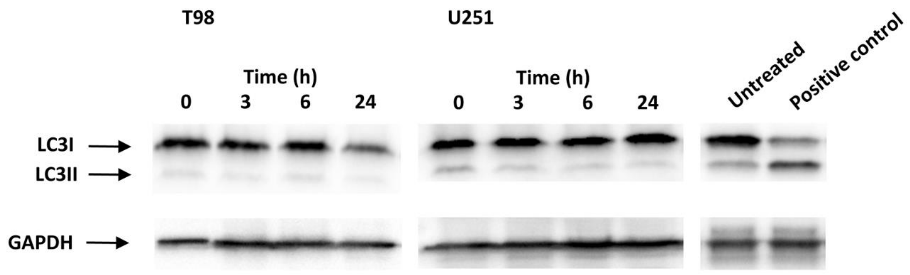

Figure S5. MK6-83 does not induce autophagy in GBM cell lines. Lysates from T98 and U251 cells treated for 24 h with 25 and 78 μM of MK6-83, respectively, were separated on 14% SDS-PAGE and probed with anti-LC3 and anti-GAPDH Abs. GAPDH protein levels were evaluated as loading control. Blots are representative of one of three separate experiments.

Table S1. GBM (grade IV) patient characteristics.

**Patients Characteristics**	**% of samples (*n*)**
**Number of patients**	**66**
**Sex**	
Men	47% (*n* = 31)
Women	53% (*n* = 35)
**Age, years**	
< 45	55% (*n* =36)
>45	45% (*n* = 30)
**GBM MGMT methylation**	
Non detected	32% (*n* = 21)
Methylated	36% (*n* = 24)
Non methylated	32% (*n* = 21)
**GBM Recidivated**	
Not detected	9% (*n* = 6)
Not recidivated	14% (*n* = 9)
Recidivated	77% (*n* = 51)
**Adjuvant therapy**	
No	13% (*n* = 9)
STUPP	56% (*n* = 37)
Fotemustine	8% (*n* = 5)
RT	11% (*n* = 7)
Other:	12% (*n* = 8)
-Temozolomide	38% (3/8)
-Fotemustine + Bevacizumab	38% (3/8)
-Fotemustine + RT	12% (1/8)
-Fotemustine + RT + Bevacizumab	12% (1/8)

Clinicopathological characteristics of the 66 GBM patients analyzed for TRPML-1 mRNA and protein expression. Stupp protocol comprises: radiotherapy (total 60 Gy, 2 Gy per daily fraction over 6 weeks); temozolomide (during radiotherapy: 75 mg per square meter of body-surface area per day, 7 days per week; post-radiotherapy (adjuvant): six cycles consisting of 150-200 mg per square meter for 5 days during each 28-day cycle). RT, radiation therapy.

© 2019 by the authors. Licensee MDPI, Basel, Switzerland. This article is an open access article distributed under the terms and conditions of the Creative Commons Attribution (CC BY) license (http://creativecommons.org/licenses/by/4.0/).
